# Taxonomic position of several enigmatic Polyommatus (Agrodiaetus) species (Lepidoptera, Lycaenidae) from Central and Eastern Iran: insights from molecular and chromosomal data

**DOI:** 10.3897/CompCytogen.v8i4.8939

**Published:** 2014-12-09

**Authors:** Vladimir A. Lukhtanov, Nazar A. Shapoval, Alexander V. Dantchenko

**Affiliations:** 1 Department of Karyosystematics, Zoological Institute of Russian Academy of Sciences, Universitetskaya nab. 1, 199034 St. Petersburg, Russia; 2 Department of Entomology, Faculty of Biology, St. Petersburg State University, Universitetskaya nab. 7/9, 199034 St. Petersburg, Russia

**Keywords:** *Agrodiaetus*, chromosome number, *COI*, karyotype, Lycaenidae, *Polyommatus*

## Abstract

The species-rich subgenus Polyommatus (Agrodiaetus) has become one of the best studied groups of Palearctic blue butterflies (Lepidoptera, Lycaenidae). However, the identity and phylogenetic position of some rare taxa from Iran have remained unclear. An enigmatic, recently described Central Iranian species Polyommatus (Agrodiaetus) shirkuhensis ten Hagen et Eckweiler, 2001 has been considered as a taxon closely related either to Polyommatus (Agrodiaetus) eckweileri ten Hagen, 1998 or to Polyommatus (Agrodiaetus) baltazardi (de Lesse, 1962). Polyommatus (Agrodiaetus) baltazardi, in its turn, was treated as a taxon close to Iranian-Pakistani Polyommatus (Agrodiaetus) bogra Evans, 1932. Here we used a combination of molecular and chromosomal markers to show that none of these hypotheses was true. Instead, Polyommatus (Agrodiaetus) shirkuhensis was recovered as a member of a species group close to Polyommatus (Agrodiaetus) cyaneus (Staudinger, 1899). From genetically closest species, Polyommatus (Agrodiaetus) kermansis (de Lesse, 1962), Polyommatus (Agrodiaetus) cyaneus and Polyommatus (Agrodiaetus) sennanensis (de Lesse, 1959), it differs by the wing coloration. From morphologically similar Polyommatus (Agrodiaetus) mofidii (de Lesse, 1963) and Polyommatus (Agrodiaetus) sorkhensis Eckweiler, 2003, it differs by its chromosome number, n=21. Polyommatus (Agrodiaetus) bogra and Polyommatus (Agrodiaetus) baltazardi were found to be members of two different species groups and, thus, are not closely related.

## Introduction

Agrodiaetus Hübner, 1822, a subgenus of the species-rich Palearctic genus *Polyommatus* Latreille, 1804 (Talavera et al. 2013) is a model system in studies of speciation (Lukhtanov et al. 2005, Wiemers et al. 2009), intraspecific differentiation (Dinca et al. 2013, Przybyłowicz et al. 2014), and rapid karyotype evolution (Lukhtanov and Dantchenko 2002, Kandul et al. 2007, Vershinina and Lukhtanov 2010, 2013). Despite this fact the taxonomy of the subgenus is poorly understood, and using of molecular markers in combination with cytogenetic studies resulted in recent years in discovery of new species (Lukhtanov et al. 2003, 2008) and numerous taxonomic and nomenclatural changes (Lukhtanov1989, Lukhtanov et al. 2006, Vila et al. 2010).

Here we use a combination of molecular mitochondrial (*COI*), molecular nuclear (*ITS2*) and nuclear chromosomal (karyotype) markers to analyze two recently described and little known taxa Polyommatus (Agrodiaetus) shirkuhensis ten Hagen et Eckweiler, 2001 (ten Hagen and Eckweiler 2001) and Polyommatus (Agrodiaetus) bogra birjandensis Eckweiler, 2003 (Eckweiler 2003) which status and taxonomic position is disputed in literature (ten Hagen and Eckweiler 2001, Skala 2002).

## Material and methods

The taxa Polyommatus (Agrodiaetus) shirkuhensis (Iran, Yazd Province, Shirkuh Mts., Deh-Bala village, 2900-3150 m, 12 July 2005, samples J299-1, J299-2 and J299-3, J302 and J304) and Polyommatus (Agrodiaetus) bogra birjandensis(Iran, South Khorasan Province, 26 km N of Birjand, 1900-2000 m, 14 July 2005, samples J305, J306, J307, J307-1, J307-2, J307-3, J307-4, J315, J318 and J319) were collected exactly in their type localities.

Fresh (not worn) adult males were used to investigate the karyotypes. After capturing a butterfly in the field, it was placed in a glassine envelope for 1-2 hours to keep it alive until we processed it. Testes were removed from the abdomen and placed into a small 0.5 ml vial with a freshly prepared fixative (ethanol and glacial acetic acid, 3:1). Then each wing was carefully removed from the body using forceps. The wingless body was placed into a plastic, 2 ml vial with pure 96% ethanol. The samples are kept in the Zoological Institute of the Russian Academy of Sciences.

Testes were stored in the fixative for 1-12 months at +4°C. Then the gonads were stained in 2% acetic orcein for 30-60 days at +18-20°C. Different stages of male meiosis were examined by using a light microscope Amplival, Carl Zeiss. We have used an original two-phase method of chromosome analysis (Lukhtanov and Dantchenko 2002, Lukhtanov et al. 2006).

A 643 bp fragment of mitochondrial gene *cytochrome oxidase subunit I* (*COI*) and 592 bp fragment of *nuclear internal transcribed spacer 2* (*ITS2*) were used to analyze clustering of the specimens. Primers and the protocol of DNA amplification were given in our previous publication (Lukhtanov et al. 2008). The sequences were edited and aligned using BioEdit 7.0.3 (Hall 1999). Since *Polyommatus icarus* (Rottemburg, 1775) and *Polyommatus stempfferi* (Brandt, 1938) were earlier inferred as outgroups to the subgenus *Agrodiaetus* (Talavera et al. 2013), we used them to root the phylograms.

Sequences of the following additional representatives of the subgenus *Agrodiaetus* were found in GenBank (Wiemers 2003, Wiemers and Fiedler 2007, Wiemers et al. 2009, Kandul et al. 2004, 2007, Lukhtanov et al. 2005) and used for phylogenetic inference: Polyommatus (Agrodiaetus) ainsae (Forster, 1961), Polyommatus (Agrodiaetus) achaemenes Skala, 2002, Polyommatus (Agrodiaetus) actinides (Staudinger, 1886), Polyommatus (Agrodiaetus) admetus malievi (Dantchenko et Lukhtanov, 2005), Polyommatus (Agrodiaetus) aereus Eckweiler, 1998, Polyommatus (Agrodiaetus) alcestis karacetinae (Lukhtanov et Dantchenko, 2002), Polyommatus (Agrodiaetus) altivagans (Forster, 1956), Polyommatus (Agrodiaetus) antidolus (Rebel, 1901), Polyommatus (Agrodiaetus) ardschira (Brandt, 1938), Polyommatus (Agrodiaetus) baltazardi (de Lesse, 1963), Polyommatus (Agrodiaetus) baytopi (de Lesse, 1959), Polyommatus (Agrodiaetus) bilgini (Dantchenko et Lukhtanov, 2002), Polyommatus (Agrodiaetus) birunii Eckweiler et ten Hagen, 1998, Polyommatus (Agrodiaetus) caeruleus (Staudinger, 1871), Polyommatus (Agrodiaetus) carmon carmon (Herrich-Schäffer, 1851), Polyommatus (Agrodiaetus) carmon munzuricus (Rose, 1978), Polyommatus (Agrodiaetus) ciscaucasicus (Forster, 1956), Polyommatus (Agrodiaetus) cyaneus (Staudinger, 1899), Polyommatus (Agrodiaetus) dagestanicus (Forster, 1960), Polyommatus (Agrodiaetus) dagmara (Grum-Grshimaïlo, 1888), Polyommatus (Agrodiaetus) damocles (Herrich-Schäffer, 1844), Polyommatus (Agrodiaetus) damon (Dennis et Schiffermüller, 1775), Polyommatus (Agrodiaetus) damone altaicus (Elwes, 1899), Polyommatus (Agrodiaetus) damone damone (Eversmann, 1841), Polyommatus (Agrodiaetus) damone irinae (Dantchenko, 1997), Polyommatus (Agrodiaetus) dantchenkoi Lukhtanov et Wiemers, 2003, Polyommatus (Agrodiaetus) demavendi (Pfeiffer, 1938), Polyommatus (Agrodiaetus) dizinensis (Schurian, 1982), Polyommatus (Agrodiaetus) dolus vittata (Oberthür, 1892), Polyommatus (Agrodiaetus) ectabanensis (de Lesse, 1964), Polyommatus (Agrodiaetus) elbursicus (Forster, 1956), Polyommatus (Agrodiaetus) eriwanensis (Forster, 1960), Polyommatus (Agrodiaetus) erschoffii (Lederer, 1869), Polyommatus (Agrodiaetus) faramarzii Skala, 2001, Polyommatus (Agrodiaetus) femininoides (Eckweiler, 1987), Polyommatus (Agrodiaetus) firdussii (Forster, 1956), Polyommatus (Agrodiaetus) fulgens (Sagarra, 1925), Polyommatus (Agrodiaetus) glaucias (Lederer, 1870), Polyommatus (Agrodiaetus) gorbunovi (Dantchenko et Lukhtanov, 1994), Polyommatus (Agrodiaetus) haigi (Dantchenko et Lukhtanov, 2002), Polyommatus (Agrodiaetus) hamadanensis (Lesse, 1959), Polyommatus (Agrodiaetus) hopfferi (Gerhard, 1851), Polyommatus (Agrodiaetus) huberti (Carbonell, 1993), Polyommatus (Agrodiaetus) iphidamon (Staudinger, 1899), Polyommatus (Agrodiaetus) iphigenia (Herrich-Schäffer, 1847), Polyommatus (Agrodiaetus) iphigenides (Staudinger, 1886), Polyommatus (Agrodiaetus) karatavicus Lukhtanov, 1990, Polyommatus (Agrodiaetus) karindus (Riley, 1921), Polyommatus (Agrodiaetus) kendevani (Forster, 1956), Polyommatus (Agrodiaetus) kermansis (de Lesse, 1963), Polyommatus (Agrodiaetus) khorasanensis (Carbonell, 2001), Polyommatus (Agrodiaetus) klausschuriani ten Hagen, 1999, Polyommatus (Agrodiaetus) kurdistanicus (Forster, 1961), Polyommatus (Agrodiaetus) lorestanus Eckweiler, 1997, Polyommatus (Agrodiaetus) lukhtanovi (Dantchenko, 2005), Polyommatus (Agrodiaetus) luna Eckweiler, 2002, Polyommatus (Agrodiaetus) magnificus (Grum-Grshimaïlo, 1885), Polyommatus (Agrodiaetus) masulensis ten Hagen et Schurian, 2000, Polyommatus (Agrodiaetus) mediator (Dantchenko et Churkin, 2003), Polyommatus (Agrodiaetus) menalcas (Freyer, 1837), Polyommatus (Agrodiaetus) merhaba De Prins, van der Poorten, Borie, van Oorschot, Riemis et Coenen, 1991, Polyommatus (Agrodiaetus) mithridates (Staudinger, 1878), Polyommatus (Agrodiaetus) mofidii (de Lesse, 1963), Polyommatus (Agrodiaetus) ninae (Forster, 1956), Polyommatus (Agrodiaetus) peilei (Bethune-Baker, 1921), Polyommatus (Agrodiaetus) pfeifferi (Brandt, 1938), Polyommatus (Agrodiaetus) phyllides (Staudinger, 1886), Polyommatus (Agrodiaetus) phyllis (Christoph, 1877), Polyommatus (Agrodiaetus) pierceae (Lukhtanov et Dantchenko, 2002), Polyommatus (Agrodiaetus) poseidon (Herrich-Schäffer, 1851), Polyommatus (Agrodiaetus) poseidonides (Staudinger, 1886), Polyommatus (Agrodiaetus) pulcher (Sheljuzhko, 1935), Polyommatus (Agrodiaetus) putnami (Dantchenko et Lukhtanov, 2002), Polyommatus (Agrodiaetus) ripartii (Freyer, 1830), Polyommatus (Agrodiaetus) ripartii paralcestis (Forster, 1960), Polyommatus (Agrodiaetus) rjabovi (Forster, 1960), Polyommatus (Agrodiaetus) rovshani (Dantchenko et Lukhtanov, 1994), Polyommatus (Agrodiaetus) sennanensis (de Lesse, 1959), Polyommatus (Agrodiaetus) shahkuhensis (Lukhtanov, Shapoval et Dantchenko, 2008), Polyommatus (Agrodiaetus) shahrami Skala, 2001, Polyommatus (Agrodiaetus) shamil (Dantchenko, 2000), Polyommatus (Agrodiaetus) sorkhensis Eckweiler, 2003, Polyommatus (Agrodiaetus) surakovi (Dantchenko et Lukhtanov, 1994), Polyommatus (Agrodiaetus) tankeri (de Lesse, 1960), Polyommatus (Agrodiaetus) tenhageni Schurian et Eckweiler, 1999, Polyommatus (Agrodiaetus) transcaspica (Heyne, 1895), Polyommatus (Agrodiaetus) turcicolus (Koçak, 1977), Polyommatus (Agrodiaetus) turcicus (Koçak, 1977), Polyommatus (Agrodiaetus) urmiaensis Schurian et ten Hagen, 2003, Polyommatus (Agrodiaetus) vanensis sheljuzhkoi (Forster, 1960), Polyommatus (Agrodiaetus) vaspurakani (Lukhtanov et Dantchenko, 2003) and Polyommatus (Agrodiaetus) zarathustra Eckweiler, 1997.

Bayesian analysis was performed using the program MrBayes 3.2.2 (Ronquist et al. 2012). A GTR substitution model with gamma distributed rate variation across sites and a proportion of invariable sites was specified before running the program for 5,000,000 generations with default settings. The first 1250 trees (out of 5000) were discarded as a burn-in prior to computing a consensus phylogeny and posterior probabilities.

## Results

### Molecular markers

**Figure 1.**
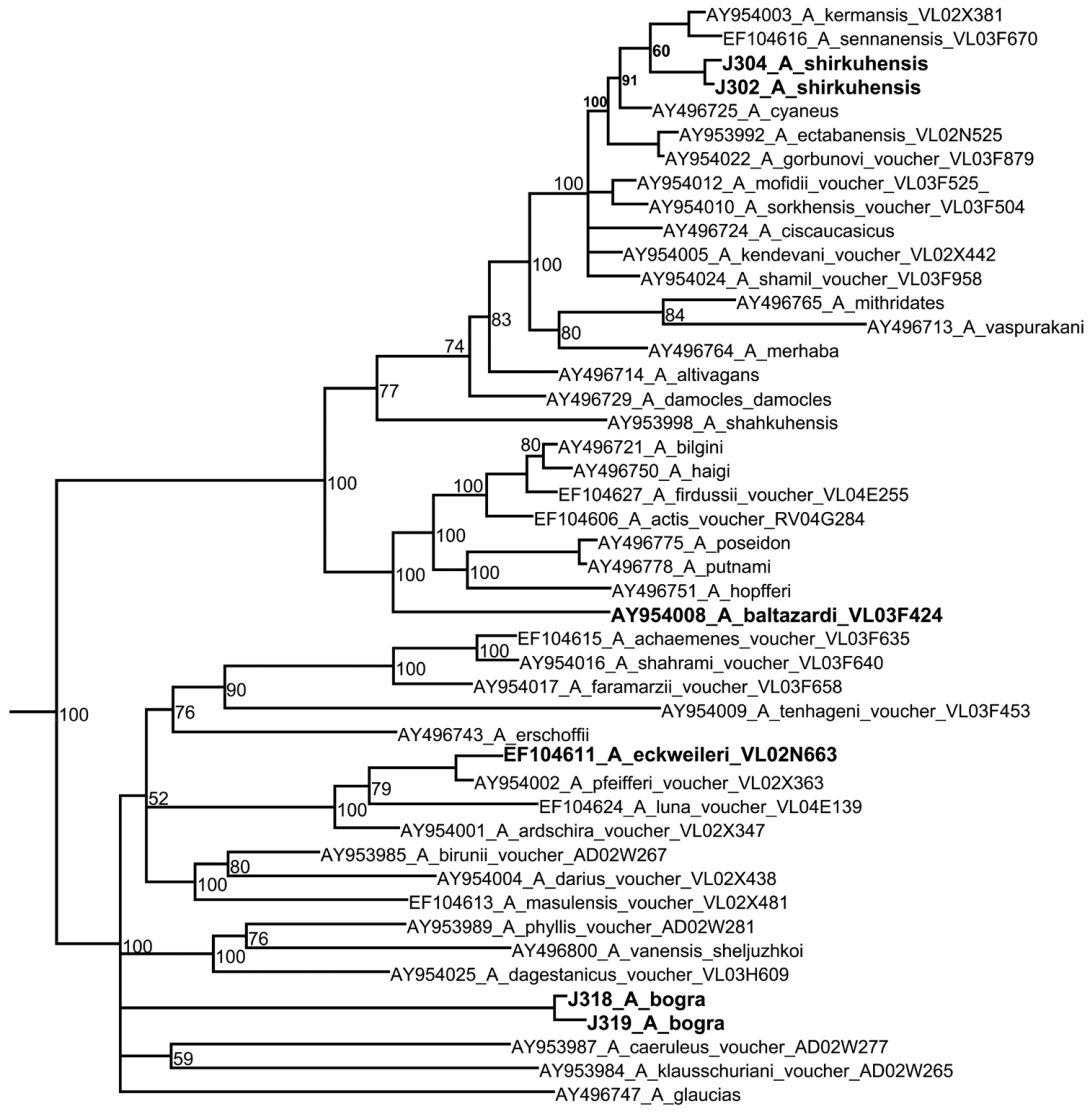
Fragment of consensus Bayesian tree of the subgenus *Agrodiaetus* inferred from *COI* sequences. Posterior probability values >50% are shown. Names of the target species are in bold. The complete tree is given online in the Suppl. material 1.

Bayesian analysis of the gene *COI* resulted in a consensus phylogram which displayed a high level of posterior probability for the majority of the clades revealed. A fragment of this tree demonstrating the position of the target species Polyommatus (Agrodiaetus) shirkuhensis, Polyommatus (Agrodiaetus) eckweileri ten Hagen, 1998, Polyommatus (Agrodiaetus) baltazardi (de Lesse, 1962) and Polyommatus (Agrodiaetus) bogra birjandensis is shown on Fig. 1.

**Figure 2.**
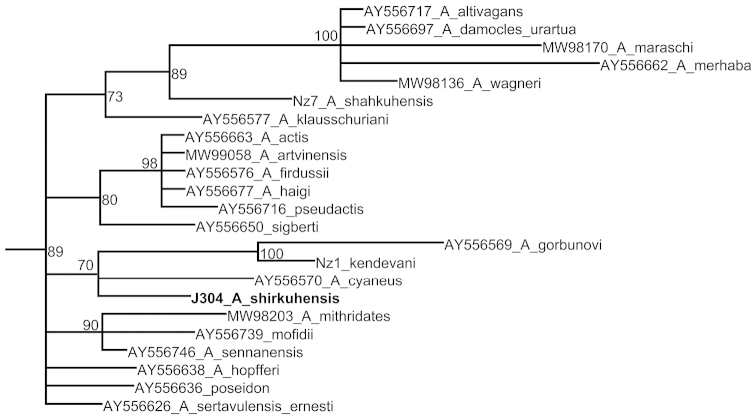
Fragment of consensus Bayesian tree of the subgenus *Agrodiaetus* inferred from *ITS2* sequences. Posterior probability values >50% are shown. Names of the target species are in bold. The complete tree is given online in the Suppl. material 2.

Bayesian analysis of the sequence *ITS2* resulted in a mostly unresolved consensus phylogram (Fig. 2), however some clades, including the clade demonstrating the position of Polyommatus (Agrodiaetus) shirkuhensis, were revealed with moderate level of posterior probability.

### Karyotypes

**Figure 3.**
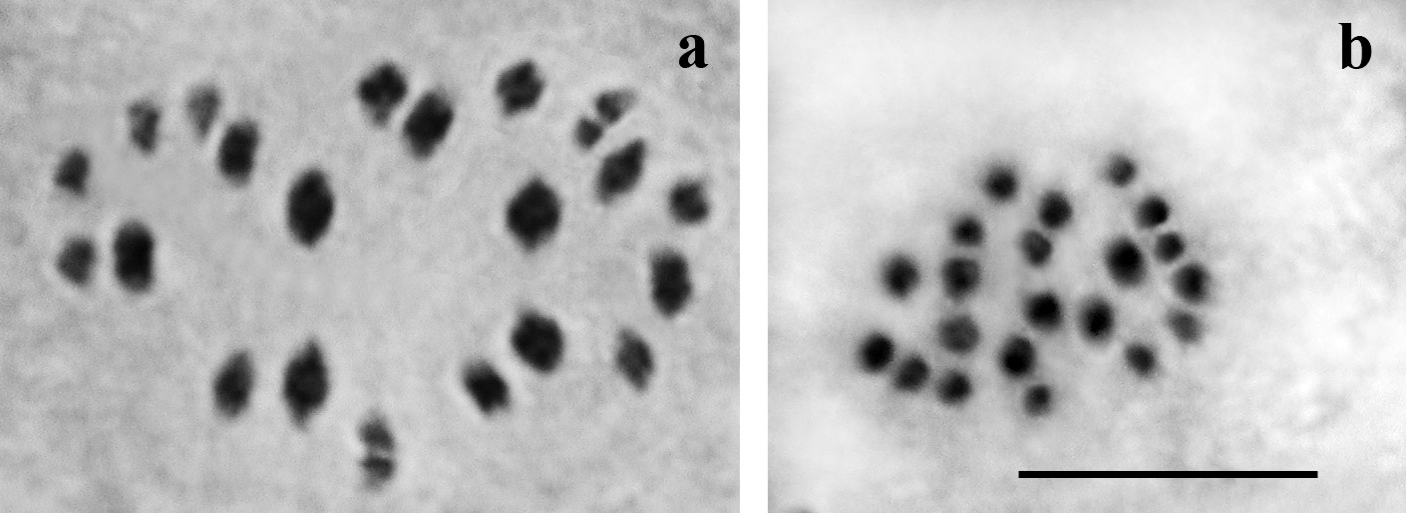
Male karyotype of Polyommatus (Agrodiaetus) shirkuhensis. **a** metaphase I, n = 21 **b** metaphase II, n = 21. Bar = 10µm.

Polyommatus (Agrodiaetus) shirkuhensis (Table 1, Fig. 3). The haploid chromosome number n=21 was found in MI and MII cells of three studied individuals (J299-1, J299-2 and J299-3). In the specimen J299-2, the number 2n=42 was found in diploid chromosome set observed in male asynaptic meiosis. In MI cells, all bivalents formed a gradient size row. The karyotype contained no exceptionally large or small bivalents.

Polyommatus (Agrodiaetus) bogra birjandensis (Table 1). Only one (J305) of nine studied specimens displayed metaphase figs acceptable for chromosome analysis. In this specimen we were able to count approximately 2n=ca105-106 in male asynaptic meiosis. The count was done with approximation due to the overlapping of some chromosomes. The diploid set included one pair of exceptionally large chromosomes. Other chromosomes formed a gradient size row.

**Table 1.** Haploid chromosome number (n) of the taxa discussed and the species groups to which these taxa belong in classifications by Eckweiler and Häuser (1997) and Kandul et al. (2004).

**Taxon**	**n**	**Species group (classification after Eckweiler and Häuser)**	**Species group (classification after Kandul et al.)**	**Reference**
Polyommatus (Agrodiaetus) baltazardi	45	Polyommatus (Agrodiaetus) erschoffii	Polyommatus (Agrodiaetus) poseidon	Lukhtanov et al. 2005
Polyommatus (Agrodiaetus) bogra birjandensis	ca52–53	Polyommatus (Agrodiaetus) erschoffii	Polyommatus (Agrodiaetus) erschoffii	This paper
Polyommatus (Agrodiaetus) cyaneus	from 18 to 20	Polyommatus (Agrodiaetus) damon	Polyommatus (Agrodiaetus) cyaneus	de Lesse 1963, Kandul et al. 2007
Polyommatus (Agrodiaetus) eckweileri	ca106	unclear	Polyommatus (Agrodiaetus) erschoffii	Kandul et al. 2007
Polyommatus (Agrodiaetus) kermansis	22	Polyommatus (Agrodiaetus) damon	Polyommatus (Agrodiaetus) cyaneus	Lukhtanov et al. 2005
Polyommatus (Agrodiaetus) mofidii	35	Polyommatus (Agrodiaetus) damon	Polyommatus (Agrodiaetus) cyaneus	Lukhtanov et al. 2005
Polyommatus (Agrodiaetus) sennanensis	28–31	Polyommatus (Agrodiaetus) dolus (Hübner, 1823)	Polyommatus (Agrodiaetus) cyaneus	Kandul et al. 2007
Polyommatus (Agrodiaetus) shirkuhensis	21	unclear	Polyommatus (Agrodiaetus) cyaneus	This paper
Polyommatus (Agrodiaetus) sorkhensis	43	Polyommatus (Agrodiaetus) damon	Polyommatus (Agrodiaetus) cyaneus	Lukhtanov et al. 2005

## Discussion

Polyommatus (Agrodiaetus) shirkuhensis is the only species of the subgenus *Agrodiaetus* known from Shirkuh Mts massif in Central Iran (province Yazd) (ten Hagen and Eckweiler 2001). Immediately after its description, it attracted attention of lepidopterists (Skala 2002) because of its unusual combination of morphological characters such as loss of the white streak on the underside of the hind wings (most important apomorphy of the subgenus *Agrodiaetus* as a whole) and exaggerated elements of the wing underside pattern. A similar wing pattern is known in three other *Agrodiaetus* species from Central and Eastern Iran: Polyommatus (Agrodiaetus) eckweileri, Polyommatus (Agrodiaetus) baltazardi and Polyommatus (Agrodiaetus) bogra Evans, 1932. From these three species, Polyommatus (Agrodiaetus) bogra has the white streak on the hind wing underside, whereas Polyommatus (Agrodiaetus) eckweileri and Polyommatus (Agrodiaetus) baltazardi do not (Eckweiler and Häuser 1997, ten Hagen and Eckweiler 2001, Skala 2002). All four species are allopatric in their distribution ranges (ten Hagen and Eckweiler 2001).

Ten Hagen and Eckweiler (2001) hypothesized that Polyommatus (Agrodiaetus) shirkuhensis was a taxon closely related either to Polyommatus (Agrodiaetus) eckweileri (distributed in province Esfahan) or to Polyommatus (Agrodiaetus) baltazardi (distributed in province Kerman). Polyommatus (Agrodiaetus) baltazardi, in its turn, was treated by them as a taxon close to East Iranian – Pakistani species Polyommatus (Agrodiaetus) bogra.

However, analysis of *COI* clusters in the Bayesian tree (Fig. 1) showed that none of these hypotheses was true. Among the major species groups recognized within the subgenus *Agrodiaetus* by Kandul et al. (2004, 2007) (Table 1), Polyommatus (Agrodiaetus) eckweileri is recovered by us as a member of *Polyommatus pfeifferi* (Brandt, 1938) – *Polyommatus ardschira* (Brandt, 1938) – *Polyommatus luna* Eckweiler, 2002 species complex belonging to *Polyommatus erschoffii* (Lederer, 1869) group.

Polyommatus (Agrodiaetus) baltazardi is found to be a member of Polyommatus (Agrodiaetus) poseidon (Herrich-Schäffer, [1851]) group and, thus, is not related to Polyommatus (Agrodiaetus) bogra The latter species has very isolated position within the *Polyommatus erschoffii* group. The karyotypes of Polyommatus (Agrodiaetus) baltazardi and Polyommatus (Agrodiaetus) bogra are also different (Table 1).

Finally, our target species, Polyommatus (Agrodiaetus) shirkuhensis, is found to be a member of Polyommatus (Agrodiaetus) cyaneus (Staudinger, 1899) group and is especially close to Polyommatus (Agrodiaetus) kermansis (de Lesse, 1962), Polyommatus (Agrodiaetus) sennanensis (de Lesse, 1959) and Polyommatus (Agrodiaetus) cyaneus (Fig. 1). The position of Polyommatus (Agrodiaetus) shirkuhensis on the *ITS2* tree (Fig. 2) also does not contradict the conclusion that Polyommatus (Agrodiaetus) shirkuhensis belongs to Polyommatus (Agrodiaetus) cyaneus species group.

From Polyommatus (Agrodiaetus) kermansis, Polyommatus (Agrodiaetus) cyaneus and Polyommatus (Agrodiaetus) sennanensis, which possess closest *COI* haplotypes, Polyommatus (Agrodiaetus) shirkuhensis differs by blue upper side of wings in males (it is deep violet in Polyommatus (Agrodiaetus) kermansis, violet in Polyommatus (Agrodiaetus) cyaneus and whitish in Polyommatus (Agrodiaetus) sennanensis) (see figures in Eckweiler and Häuser 1997). The wing color in Polyommatus (Agrodiaetus) shirkuhensis is similar to those found in Polyommatus (Agrodiaetus) mofidii (de Lesse, 1963) and Polyommatus (Agrodiaetus) sorkhensis Eckweiler, 2003 (see figs 18–25 in Eckweiler 2003), two other members of the Polyommatus (Agrodiaetus) cyaneus group. Polyommatus (Agrodiaetus) mofidii, Polyommatus (Agrodiaetus) sorkhensis and Polyommatus (Agrodiaetus) shirkuhensis are allopatric in their distribution ranges (ten Hagen and Eckweiler 2001, Eckweiler 2003) and significantly different in their karyotypes (Table 1).

To conclude, our study demonstrates that four allopatric taxa known from Central and East Iran, Polyommatus (Agrodiaetus) shirkuhensis, Polyommatus (Agrodiaetus) eckweileri, Polyommatus (Agrodiaetus) baltazardi and Polyommatus (Agrodiaetus) bogra birjandensis, which possess significant elements of morphological similarity, are not only specifically distinct from each other, but even belong to different distantly related groups of species within the subgenus *Agrodiaetus*.

## Supplementary materials

Supplementary material 1 Consensus Bayesian tree of the subgenus Polyommatus (Agrodiaetus) inferred from COI sequences Data type: image Explanation note: Consensus Bayesian tree of the subgenus *Polyommatus* (Agrodiaetus) inferred from *COI* sequences. Posterior probability values >50% are shown. Names of the target species are in bold. This dataset is made available under the Open Database License (http://opendatacommons.org/licenses/odbl/1.0/). The Open Database License (ODbL) is a license agreement intended to allow users to freely share, modify, and use this Dataset while maintaining this same freedom for others, provided that the original source and author(s) are credited.Vladimir A. Lukhtanov, Nazar A. Shapoval, Alexander V. Dantchenko

Supplementary material 2 Consensus Bayesian tree of the subgenus Polyommatus (Agrodiaetus) inferred from ITS2 sequences Data type: image Explanation note: Consensus Bayesian tree of the subgenus *Polyommatus* (Agrodiaetus) inferred from *ITS2* sequences. Posterior probability values >50% are shown. Names of the target species are in bold. This dataset is made available under the Open Database License (http://opendatacommons.org/licenses/odbl/1.0/). The Open Database License (ODbL) is a license agreement intended to allow users to freely share, modify, and use this Dataset while maintaining this same freedom for others, provided that the original source and author(s) are credited.Vladimir A. Lukhtanov, Nazar A. Shapoval, Alexander V. Dantchenko
