# Supplementary figures and images for: Taxonomic position of several enigmatic Polyommatus (Agrodiaetus) species (Lepidoptera, Lycaenidae) from Central and Eastern Iran: insights from molecular and chromosomal data
Source: Comp Cytogenet. 2014 Dec 9;8(4):313–22. doi: 10.3897/CompCytogen.v8i4.8939 (PMC4296718; doi:10.3897/CompCytogen.v8i4.8939)

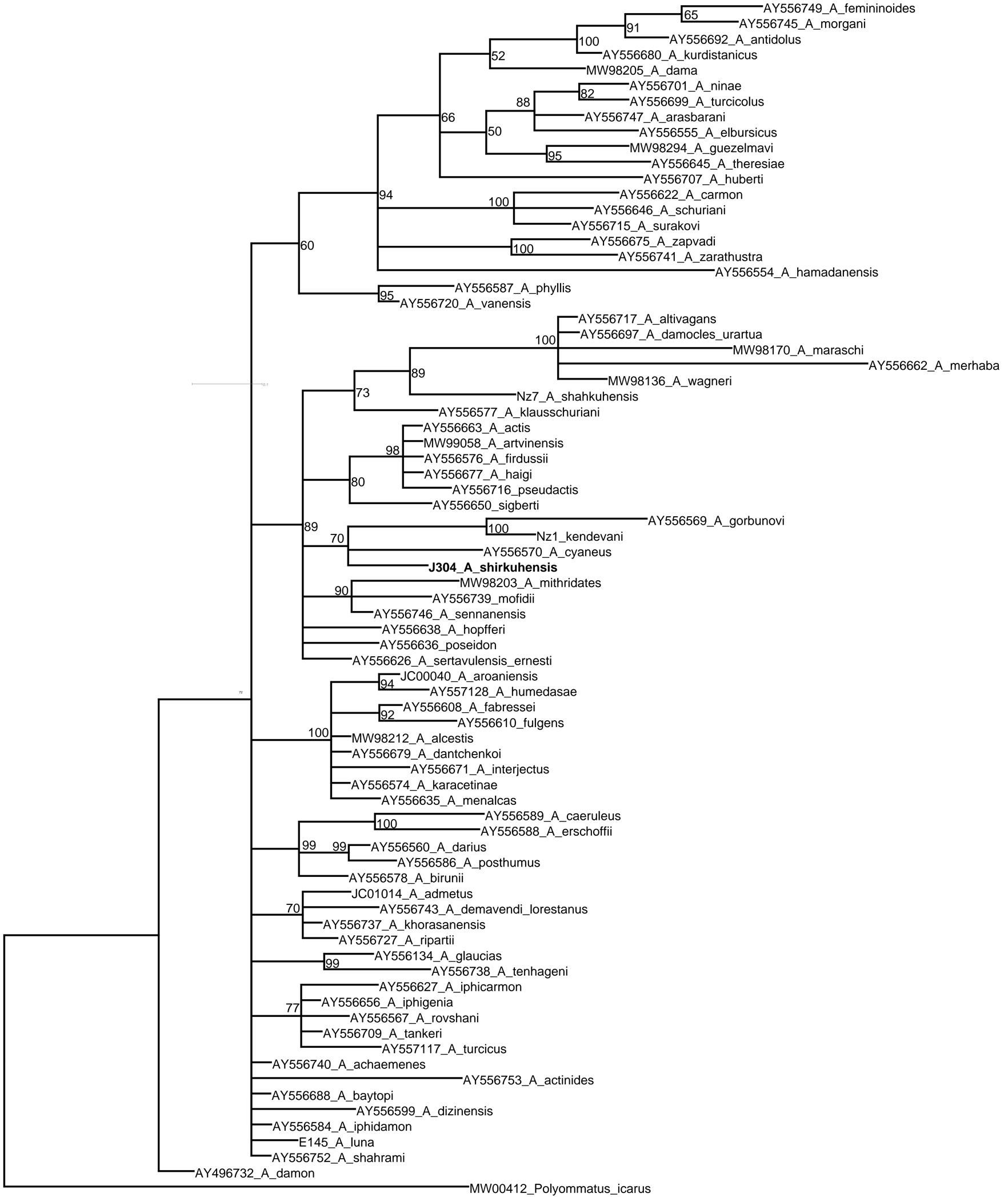

Supplement: Supplementary material 2 — Consensus Bayesian tree of the subgenus Polyommatus (Agrodiaetus) inferred from ITS2 sequences [file CompCytogen-8-313-s002.pdf]
