# Supplementary material for: Taxonomic position of several enigmatic Polyommatus (Agrodiaetus) species (Lepidoptera, Lycaenidae) from Central and Eastern Iran: insights from molecular and chromosomal data
Source: Comp Cytogenet. 2014 Dec 9;8(4):313–22. doi: 10.3897/CompCytogen.v8i4.8939 (PMC4296718; doi:10.3897/CompCytogen.v8i4.8939)
Supplement: Supplementary material 1 — Consensus Bayesian tree of the subgenus Polyommatus (Agrodiaetus) inferred from COI sequences [file CompCytogen-8-313-s001.pdf]

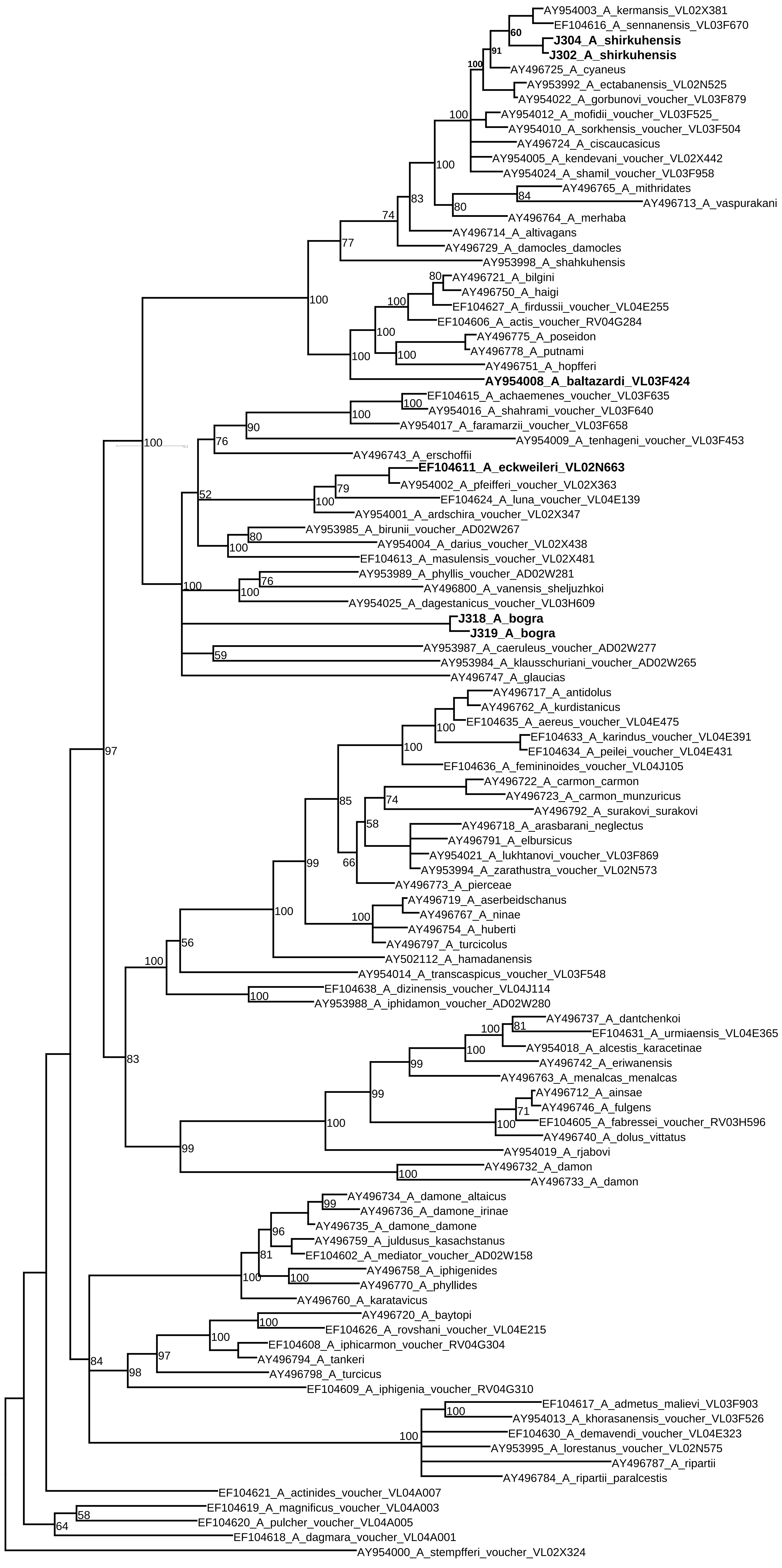
AY954003_A_kermansis_VL02X381
EF104616_A_sennanensis_VL03F670
J304_A_shirkuhensis
J302_A_shirkuhensis
AY496725_A_cyaneus
AY953992_A_ectabanensis_VL02N525
AY954022_A_gorbunovi_voucher_VL03F879
AY954012_A_mofidii_voucher_VL03F525_
AY954010_A_sorkhensis_voucher_VL03F504
AY496724_A_ciscaucasicus
AY954005_A_kendevani_voucher_VL02X442
AY954024_A_shamil_voucher_VL03F958
AY496765_A_mithridates
AY496713_A_vaspurakani
AY496764_A_merhaba
AY496714_A_altivagans
AY496729_A_damocles_damocles
AY953998_A_shahkuhensis
AY496721_A_bilgini
AY496750_A_haigi
EF104627_A_firdussii_voucher_VL04E255
EF104606_A_actis_voucher_RV04G284
AY496775_A_poseidon
AY496778_A_putnami
AY496751_A_hopfferi
AY954008_A_baltazardi_VL03F424
EF104615_A_achaemenes_voucher_VL03F635
AY954016_A_shahrami_voucher_VL03F640
AY954017_A_faramarzii_voucher_VL03F658
AY954009_A_tenhageni_voucher_VL03F453
AY496743_A_erschoffii
EF104611_A_eckweileri_VL02N663
AY954002_A_pfeifferi_voucher_VL02X363
EF104624_A_luna_voucher_VL04E139
AY954001_A_ardschira_voucher_VL02X347
AY953985_A_birunii_voucher_AD02W267
AY954004_A_darius_voucher_VL02X438
EF104613_A_masulensis_voucher_VL02X481
AY953989_A_phyllis_voucher_AD02W281
AY496800_A_vanensis_sheljuzhkoi
AY954025_A_dagestanicus_voucher_VL03H609
J318_A_bogra
J319_A_bogra
AY953987_A_caeruleus_voucher_AD02W277
AY953984_A_klausschuriani_voucher_AD02W265
AY496747_A_glaucias
AY496717_A_antidolus
AY496762_A_kurdistanicus
EF104635_A_aereus_voucher_VL04E475
EF104633_A_karindus_voucher_VL04E391
EF104634_A_peilei_voucher_VL04E431
EF104636_A_femininoides_voucher_VL04J105
AY496722_A_carmon_carmon
AY496723_A_carmon_munzuricus
AY496792_A_surakovi_surakovi
AY496718_A_arasbarani_neglectus
AY496791_A_elbursicus
AY954021_A_lukhtanovi_voucher_VL03F869
AY953994_A_zarathustra_voucher_VL02N573
AY496773_A_pierceae
AY496719_A_aserbeidschanus
AY496767_A_ninae
AY496754_A_huberti
AY496797_A_turcicolus
AY502112_A_hamadanensis
AY954014_A_transcaspicus_voucher_VL03F548
EF104638_A_dizinensis_voucher_VL04J114
AY953988_A_iphidamon_voucher_AD02W280
AY496737_A_dantchenkoi
EF104631_A_urmiaensis_VL04E365
AY954018_A_alcestis_karacetinae
AY496742_A_eriwanensis
AY496763_A_menalcas_menalcas
AY496712_A_ainsae
AY496746_A_fulgens
EF104605_A_fabressei_voucher_RV03H596
AY496740_A_dolus_vittatus
AY954019_A_rjabovi
AY496732_A_damon
AY496733_A_damon
AY496734_A_damone_altaicus
AY496736_A_damone_irinae
AY496735_A_damone_damone
AY496759_A_juldusus_kasachstanus
EF104602_A_mediator_voucher_AD02W158
AY496758_A_iphigenides
AY496770_A_phyllides
AY496760_A_karatavicus
AY496720_A_baytopi
EF104626_A_rovshani_voucher_VL04E215
EF104608_A_iphicarmon_voucher_RV04G304
AY496794_A_tankeri
AY496798_A_turcicus
EF104609_A_iphigenia_voucher_RV04G310
EF104617_A_admetus_malievi_VL03F903
AY954013_A_khorasanensis_voucher_VL03F526
EF104630_A_demavendi_voucher_VL04E323
AY953995_A_lorestanus_voucher_VL02N575
AY496787_A_ripartii
AY496784_A_ripartii_paralcestis
EF104621_A_actinides_voucher_VL04A007
EF104619_A_magnificus_voucher_VL04A003
EF104620_A_pulcher_voucher_VL04A005
EF104618_A_dagmara_voucher_VL04A001
AY954000_A_stempfferi_voucher_VL02X324
